# Expression of Survivin, CK7, ASH1, HMGB3, L587S, and CLCA2 in Peripheral Blood of Lung Cancer Patients by Real-Time Polymerase Chain Reaction

**DOI:** 10.7759/cureus.64386

**Published:** 2024-07-12

**Authors:** Sweety Gupta, Manoj Gupta, Bela Goyal, Shashi Ranjan Mani Yadav, Anissa A Mirza, Amit Gupta, Shalinee Rao, Kusum Kumari, Siddhartha Nanda, Mrinalini Kotru

**Affiliations:** 1 Radiation Oncology, All India Institute of Medical Sciences, Rishikesh, Rishikesh, IND; 2 Biochemistry, All India Institute of Medical Sciences, Rishikesh, Rishikesh, IND; 3 General Surgery, All India Institute of Medical Sciences, Rishikesh, Rishikesh, IND; 4 Pathology, All India Institute of Medical Sciences, Rishikesh, Rishikesh, IND; 5 Nursing, All India Institute of Medical Sciences, Deoghar, Deoghar, IND; 6 Radiation Oncology, All India Institute of Medical Sciences, Raipur, Raipur, IND; 7 Pathology, University College of Medical Sciences, New Delhi, IND

**Keywords:** polymerase chain reaction, prognosis, lung cancer, expression, gene

## Abstract

Introduction

The objective of the present study was to identify gene expression in peripheral blood by a real-time polymerase chain reaction (PCR) technique in patients who have lung carcinoma.

Material and methods

Peripheral blood samples of patients with non-small cell and small cell lung cancer were collected. Target genes included survivin, CK7, ASH1, HMGB3, L587S, and CLCA2. β-Actin was the reference gene. If the mean C_T _(threshold cycle) value for a target gene is ≥40, the gene expression is considered undetectable.

Results

Fifty patients with lung carcinoma were included and 30 healthy controls. Out of the six genes, survivin showed 26.8 times fold change as compared to controls; ASH1 and L587S were 0.54 and 0.06, respectively; and HMGB3, CLCA2, and CK7 had non-significant fold change in comparison to controls. The overall detection rate of the six target genes examined in lung cancer was 84%, with 42 out of 50 patients testing positive. Higher stages and ASH1 (p = 0.031), CK7 (p = <0.001), and HMGB3, p = 0.011 were associated significantly. CLCA2 had higher expression in patients without adrenal metastases (p = 0.044).

Conclusions

Lifestyle and geographical variation might be a probable cause of variable gene expression as compared to other studies. However, further research is needed to determine the clinical implication of these markers, especially in larger groups of early-stage patients.

## Introduction

Lung cancer is mostly associated with many environmental variables, with smoking being the most significant [1]. Lung cancer has been classified by the World Health Organization (WHO) into two categories: non-small cell lung cancer (NSCLC) and small cell lung cancer (SCLC). This classification is based on the biological characteristics of the cancer, the treatment, and prognosis. Non-NSCLC (NSCLC) comprises over 85% of all lung cancer cases and consists of two primary subtypes: adenocarcinoma and squamous cell carcinoma [2]. Diagnosis is done by clinical examination for chest findings, any peripheral lymphadenopathy, radiological imaging chest X-ray, contrast-enhanced computed tomography (CECT) scan, and histopathology [3]. The tissue collection process is an invasive procedure and is limited by sensitivity and specificity. Immunohistochemistry and molecular testing are being developed for the identification of subtypes and tissue of origin as the lung. Circulating tumor cells (CTCs) were initially detected in 1869 by an Australian physician Thomas Ashworth in a breast cancer patient’s blood [4]. They are shed from primary tumor masses and deposited at other sites through the bloodstream. They have been detected in a majority of epithelial cancers, including those from breast, prostate, lung, and colon [5-8]. CTC analyses are considered a real-time “liquid biopsy” for patients with cancer. CTC enrichment is usually done by density gradient centrifugation (Ficoll-Hypaque separation) and immunomagnetic and size filtration procedures, and analysis is done by employing flow cytometry and polymerase chain reaction (PCR) [9]. CTCs have demonstrated utility in the assessment of prognosis, response assessment, and surveillance of patients [10]. CTCs have also been anticipated as surrogate biomarkers in the selection of neoadjuvant and adjuvant therapy, detection of recurrent disease, and pharmacodynamic biomarkers of novel therapeutics. Various gene markers are utilized for the detection of CTCs. Achaete-scute homologue 1 (ASH 1) gene expression in normal and lung cancer is restricted to cells having neuroendocrine features (e.g., small cell carcinoma and carcinogens) [11]. Calcium-activated chloride channel regulator 2 (CLCA2) gene, initially identified in human lungs, mammary tissue, and trachea, modulates chloride current across the plasma membrane in a calcium-dependent manner and cell adhesion [12]. It regulates cancer cell migration, invasion, and apoptosis. Loss of this gene portends a poor prognosis. It is overexpressed in squamous cell carcinoma lung (SCC). High mobility group box 3 (HMGB3) is an affiliate of the family of high mobility group architectural genes, which also includes HMGB1, HMGB2, HMGB3, and HMGB4 [13]. It is linked to carcinogenesis in lung, breast, esophageal, colon, and leukemia and is essential for the transcription, replication, recombination, and repair of DNA. Cell cytokeratin (CK) is a keratinocyte-based, differentiated cytoskeletal protein that is mainly dispersed in epithelial cells and is involved in the integration, continuity, and differentiation of epithelial tissues [14]. Survivin is an inhibitor of an apoptosis protein family, spans 14.7 kb at the telomeric end of chromosome 17, and is expressed in many cancers [15]. Nearly all cancers have a different survivin expression profile compared to normal tissues, and it is one of the vital genes involved in tumor aggressiveness and therapy resistance. L587S, a novel gene, is located on chromosome 18 [16]. It was identified through subtraction libraries to be specific to lung cancer. The purpose of this research was to study the expression of genes in peripheral blood by real-time polymerase chain reaction in patients of carcinoma lung and the association of these markers with clinicopathological staging and other parameters.

## Materials and methods

Patient selection

Newly diagnosed patients of NSCLC, SCLC, and healthy controls were included in the present study. Approval to conduct the study was obtained from the Institutional Ethical Committee (All India Institute of Medical Sciences, Rishikesh), and written informed consent was taken from all patients. All the patients underwent tissue biopsy, CECT thorax and abdomen, a bone scan, and a PET CT scan as per the requirement. The staging was done according to the criteria of the American Joint Commission on Cancer 8th Edition (AJCC). Patients with synchronous or metachronous second malignancy were excluded.

Processing of blood samples

Specifically, 7 mL peripheral blood (PB) samples were collected from each patient and control. Mononuclear lymphocyte cells were isolated by ficolle density gradient centrifugation, effectively excluding red blood cells and serum. Tissue collection for the positive control was obtained from a patient diagnosed with lung cancer.

RNA extraction

Isolated peripheral mononuclear blood cells were dissolved in Trizol reagent, and RNA was extracted. The quality of extracted RNA was accessed using Qubit 4 fluorometer (Thermo Fisher Scientific, Waltham, MA), and the 260/280 ratio (260/280 ratio > 0.8) and further integrity were checked on agarose gel electrophoresis. Isolated RNA was stored at -80 degrees C for further use.

cDNA synthesis

First-strand cDNA was synthesized from total RNA using a cDNA first-strand synthesis kit. cDNA synthesis was confirmed on agarose gel electrophoresis.

Real-time PCR

The primers of target genes survivin, CK-7, ASH1, HMGB3, L587S, and CLCA2 were designed, and the sequence was analyzed on primer blast and synthesized by IDT Gene Biotechnology (IDT, England). Sequences of primers are detailed in Table 1. 

**Table 1:** Sequences of primers for target and reference genes

Genes	Forward Primer	Reverse Primer	Product Size	
HMGB3	GGCCACCGTCTGGATTCTTC	TCAGCAACATCCTTCT	193bp	
CLCA2	ATGGCAGAGGCTGACAGACTC	TTCAACCACCTCAAATCCTTTCTTA	249bp	
ASH1	CCGACACGAGAAAGATGCTG	GTACTGATGGCAGAAGGAC	190bp	
Survivin	GGACCACCGCATCTCTACAT	GTTCCTCTATGGGGTCGTCATC	185bp	
CK7	CGACAACATCAAGAACCAGCGTG	GGTAGGTGGCGATCTCGATG	212bp	
L587S	CCCATTTGCCTCAAGTAACAG	GTACGAAGGAACACCATTGAAC	176bp	
β-Actin	ACTGGAACGGTGAAGGTGAC	AGAGAAGTGGGGTGGCTTTT	169bp	

The housekeeping gene, β-actin, was used as a reference gene to normalize the expression levels. The mRNA levels of genes of interest were measured by real-time PCR (CFX96 Real-Time; Bio-Rad Laboratories, Hercules, CA) using SYBR Green master mix (KAPA SYBR fast qPCR Master Mix (2X) kit). Optimal annealing temperatures were determined by gradient PCR (Table 2).

**Table 2:** Real-time PCR amplification condition for target genes

Target Gene	Pre-denaturation	Denaturation	Annealing	Extension	No. of Cycles
Survivin	95°C for 5 min	95°C for 30 sec	62°C for 30sec	70°C for 30sec	40
CK7	95°C for 5 min	95°C for 30 sec	54°C for 30sec	70°C for 27sec	40
L587S	95°C for 5 min	95°C for 30 sec	56°C for 30sec	70°C for 27sec	40
HMGB3	95°C for 5 min	95°C for 30 sec	56°C for 30sec	72°C for 27sec	40
CLCA2	95°C for 5 min	95°C for 30 sec	56°C for 30sec	72°C for 27sec	40
ASH1	95°C for 5 min	95°C for 30 sec	52°C for 30sec	72°C for 30sec	40

The qPCR reaction was conducted in a final volume of 10 uL using SYBR Green PCR Master mix in duplicate, along with a no-template control (NTC). Duplicate testing was conducted on all genes to verify the repeatability of the results. Specificity was verified by the use of melt curve analysis.

Data analysis

Gene expression levels were measured by quantitative real-time PCR software. The concentration of each target gene survivin, CK-7, ASH1, HMGB3, L587S, and CLCA2 was normalized against that of β-actin in each sample (relative quantification utilized).

(i) Normalize CT (target gene) to CT (reference gene)

 for control sample = 2 ( ∆CT (target control) - ∆CT (reference control))

 for test sample = 2 ( ∆CT (target test) - ∆CT (reference test))

(ii) Gene fold change = 2 ( ∆CT (target test) - ∆CT (reference test)) / 2 ( ∆CT (target control) - ∆CT (reference control))

Statistical analysis

A dot plot was plotted to compare the expression of survivin, CK-7, ASH1, HMGB3, L587S, and CLCA2 in lung cancer patients and controls. Expression of survivin, CK-7, ASH1, HMGB3, L587S, and CLCA2 in lung cancer patients was then sub-grouped based on different stages, histological subtypes, and patients' smoking status and gender. A Mann-Whitney U test was applied to compare the difference in expression amongst different groups. Positive expression of the six markers and clinicopathological features were examined using chi-square and Fisher's exact tests. P<0.05 was considered a statistically significant difference.

## Results

Patient and healthy control demographics

Fifty patients with newly diagnosed lung carcinoma were included and 30 healthy controls. Age ranged from 31 to 77 years (mean: 58). Healthy controls age ranged from 32 to 62 (mean: 45) years. The sociodemographic profile of patients is given in Table 3.

**Table 3:** Sociodemographic profile of patients with lung carcinoma

Clinicopathological Details	Number (n=50)
Age	31-77 years (mean: 58)
Male:female	9:1
Smokers	46 (92%)
Non-smokers	04 (08%)
Non-small cell carcinoma	42 (84%)
Adenocarcinoma	21 (50%)
Squamous carcinoma	20 (47.6%)
Not specified (NSCLC)	01 (2.38%)
Small cell carcinoma	08 (16%)
Stage III	17 (34%)
Stage IV	33 (66%)
Brain metastases	05 (15.1%)
Bone metastases	18 (54.5%)
Liver metastases	10 (30.3%)
Adrenal metastases	03 (9.09%)

Relative expression of target genes in lung cancer patients

**Figure 1:**
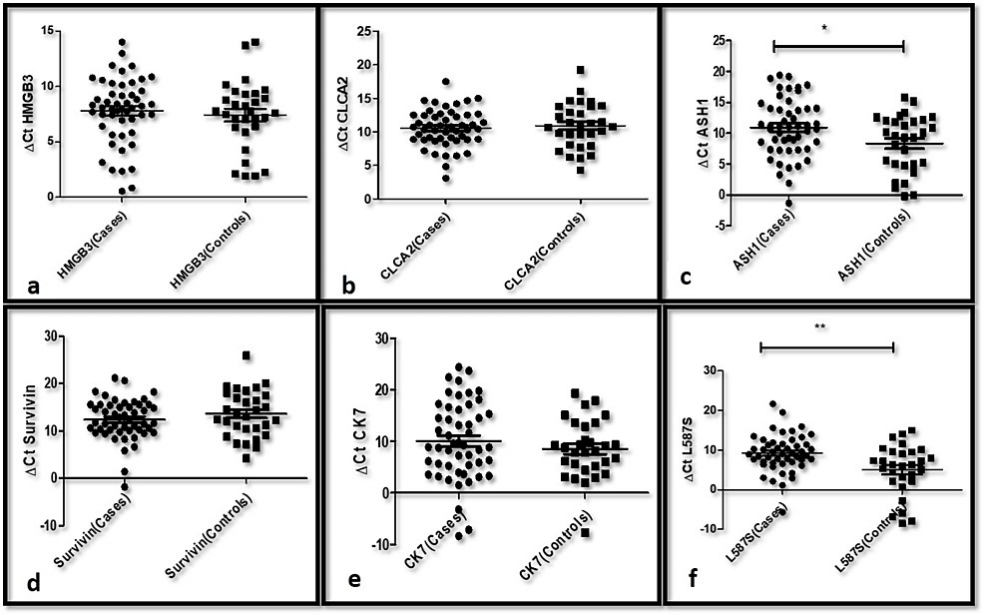
Dot plots of ΔCt values of gene expression in lung cancer patients compared to controls (a) HMGB3; (b) CLCA2; (c) ASH1; (d) Survivin; (e) Ck7; (f) L587S

The ΔCt values of HMGB3 in lung cancer patients were 0.55-14.02 (mean: 7.794) and 1.94-14.02 (mean: 7.742) in healthy controls. The ΔCt values of CLCA2 in lung cancer patients were 3.99-17.52 (mean: 10.55) and 1.94-14.02 (mean: 7.742) in healthy controls. The ΔCt values of ASH1 in lung cancer patients were -1.307-19.4 (mean: 10.93) and -0.196-15.85 (mean: 8.34) in healthy controls. The ΔCt values of survivin in lung cancer patients were 4.21-25.9 (mean: 13.6) and -8.36 -10.1 (mean:10.1) in healthy controls. The ΔCt values of Ck7 in lung cancer patients were -8.36-24.42 (mean: 10.1) and -7.67-19.37 (mean: 8.52) in healthy controls. The ΔCt values of L587S in lung cancer patients were -5.63-21.69 (mean: 9.241) and -8.49-15.01 (mean: 5.03) in healthy controls. The dot plot of all the six target genes has been illustrated in Figure 1.

Expression rates of target genes in lung cancer patients

**Figure 2:**
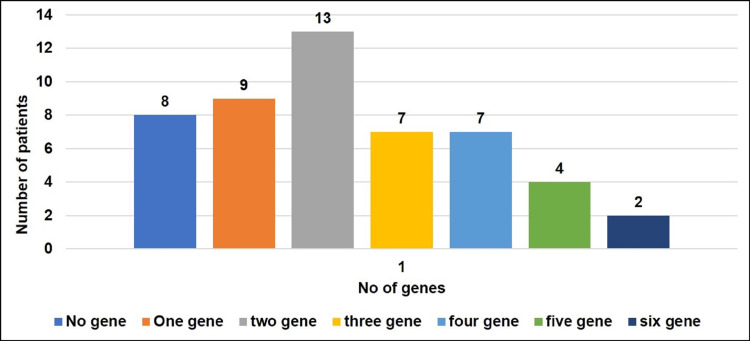
Distribution of patients according to the number of gene expression

The total positive detection rate for six target genes in lung cancer patients was 42 (84%), with two genes being the most common in 13 (26%), one gene in nine (18%), and three and four genes in seven (14%) each. On the contrary, in eight (16%) lung cancer patients, none of the target genes were detected in the PB samples (Figure 2).

**Figure 3:**
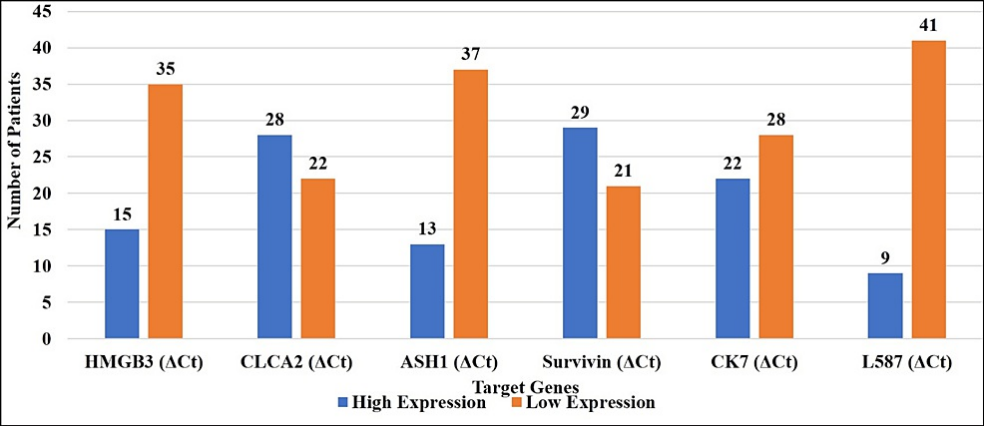
High and low expressions of target genes in lung cancer patients

The individual positive detection rate for each gene was as follows: HMGB3 of 15 (30%); CLCA2 of 28 (56%); ASH1 of 13 (26%); Survivin of 29 (58%); Ck7 of 22 (44%); and L587S of nine (18%) (Figure 3).

In adenocarcinoma histology, the highest expression was of CLCA2 in 13 (62%), followed by survivin in 12 (57.1%) patients. L587S was the least in four (19%) patients. In squamous cell carcinoma histology, survivin was most commonly expressed in 11 (55%) patients, followed by CLCA2 and CK7 in nine (45%) patients each. In this histology, the L587S gene was also least expressed in two (10%) patients. In small cell carcinoma histology, survivin was the most commonly expressed in six (80%) patients. L587S was more commonly expressed in small cell histology in three (37.5%) as compared to NSCLC. The least expressed gene was ASH1 in two (25%) patients.

Association of target genes with age, gender, smoking, stage, and histology

Target gene expression levels were analyzed to identify an association with the clinicopathological variables. In patients aged more than 50 years, CLCA2 was positive in 24 patients, while in ages less than 50 years, HMGB3 was positive in three patients. There was no statistically significant association between age and HMGB3 (χ²(1) = 0.24, p = 0.92) and ASH1 (χ²(1) = 1.343, p = 0.246), but it was significant with CLCA2 (χ²(1) = 5.128, p = 0.023), survivin (χ²(1) = 4.5, p = 0.033), and CK7 (χ²(1) = 4.687, p = 0.030). The most common gene expression in males was survivin in 25 patients, followed by CLCA2 in 27 patients, whereas in females, only survivin was expressed in four patients. There was no statistically significant association between gender and HMGB3 (χ²(1) = 0.26, p = 0.607), CLCA2 (χ²(1) = 2.922, p = 0.087), ASH1 (χ²(1) = 1.952, p = 0.162), survivin (χ²(1) = 1.103, p = 0.293), CK7 (χ²(1) = 1.103, p = 0.293), and L587S (χ²(1) = 0.015, p = 0.902). In smokers, survivin and CLCA2 were positive in 28 and 27 patients, respectively. There was no statistically significant association between smoking and HMGB3 (χ²(1) = 0.05, p = 0.82), CLCA2 (χ²(1) = 1.7, p = 0.193), survivin (χ²(1) = 1.94, p = 0.163), and CK7 (χ²(1) = 0.64, p = 0.425). In stage III, CK7 was positive in 13 patients, whereas survivin was more positive in 17 patients in stage IV. There was statistically significant relationship between stage IV and ASH1 (χ²(1) = 4.641, p = 0.031), CK7 (χ²(1) = 9.627, p = <0.001), and HMGB3 (χ²(1) = 6.46, p = 0.011), but not with CLCA2 (χ²(1) = 1.675, p = 0.195), survivin (χ²(1) = 1.44, p = 0.23), and L587S (χ²(1) = 0.15, p = 0.693; Table 4). According to histology, survivin was positive in 25 patients of NSCLC, followed by CLCA2 in 23 patients. Similarly in the SCLC gene, CLCA2 was positive in five patients, followed by survivin and CK7 in four patients each. There was no statistically significant relationship between NSCLC/SCLC and HMGB3 (χ²(1) = 0.26, p = 0.607), CLCA2 (χ²(1) = 0.04, p = 0.849), ASH1 (χ²(1) = 0.1, p = 0.747), survivin (χ²(1) = 1.1, p = 0.293), CK7 (χ²(1) = 0.04, p = 0.849), and L587S (χ²(1) = 1.82, p = 0.177; Table 4).

**Table 4:** Association of target genes with age, gender, smoking, stage, and histology Fischer’s test was used in the cell with a frequency of less than 5. *: Statistically significant, NA: Not applicable

	HMGB3		χ² p-value	CLCA2		p-value	ASH1		χ² p-value	Survivin		χ² p-value	CK7		χ² p-value	L587S		χ² p-value
Characteristics	Positive	Negative		Positive	Negative		Positive	Negative		Positive	Negative		Positive	Negative		Positive	Negative	
Gender																		
Male (45)	13 (28.8%)	32 (71.2%)	0.26, 0.607	27 (60%)	18 (40%)	2.922 0.087	13 (28.8%)	32 (71.2%)	1.952, 0.162	25 (55.5%)	20 (44.5%)	1.103, 0.293	20 (44.5%)	25 (55.5%)	1.103, 0.293	8 (17.7%)	37 (82.2%)	0.015, 0.902
Female (5)	2 (40%)	3 (60%)		1 (20%)	4 (80%)		0 (0%)	5 (100%)		4 (80%)	1 (20%)		1 (20%)	4 (80%)		1 (20%)	4 (80%)	
Age																		
≤50 years (10)	3 (30%)	7 (70%)	0.24, 0.92	2 (20%)	8 (80%)	5.128, 0.023*	1 (10%)	9 (90%)	1.343, 0.246	2 (20%)	8 (80%)	4.5, 0.033	1 (10%)	9 (90%)	4.687, 0.030	0 (0%)	10 (100%)	NA
> 50 years (40)	12 (30%)	28 (70%)		24 (60%)	16 (40%)		11 (27.5%)	29 (72.5%)		23 (57.5%)	17 (42.5%)		19 (47.5%)	21 (52.5%)		9 (22.5%)	31 (77.5%)	
Smoking																		
Yes (46)	14 (30.4%)	32 (69.6%)	0.05, 0.820	27 (58.6%)	19 (41.4%)	1.7, 0.192	13 (67.4%)	33 (23.6%)	NA	28 (60.8%)	18 (39.2%)	1.94, 0.163	21 (45.6%)	25 (54.4%)	0.64, 0.424	9 (19.5%)	35 (80.5%)	NA
No (4)	1 (25%)	3 (75%)		1 (25%)	3 (75%)		0 (0%)	4 (100%)		1 (25%)	3 (75%)		1 (25%)	3 (75%)		0(0%)	4 (100%)	
Stage																		
3 (17)	8 (29.5%)	9 (70.5%)	0.011	11 (64.7%)	6 (35.3%)	1.675, 0.195	6 (35.2%)	11 (64.8%)	4.641, 0.031	12 (70.5%)	5 (29.5%)	1.44, 0.23	13 (76.4%)	4 (23.6%)	9.627, 0.001	3 (17.6%)	11 (82.4%)	0.15, 0.693
4 (33)	6 (29.8%)	27 (70.2%)		17 (51.5%)	16 (48.5%)		7 (21.2%)	26 (78.8%)		17 (51.6%)	16 (48.4)		10 (29.8%)	23 (70.2%)		6 (18.1%)	30 (81.9%)	
Histology																		
NSCLC (42)	12 (28.6%)	30 (71.4%)	0.26, 0.607	23 (54.8%)	19 (45.2%)	0.04, 0.849	13 (31.9%)	29 (69.1%)	0.1, 0.747	25 (59.6%)	17 (40.4%)	1.1, 0.293	18 (42.8%)	24 (57.2%)	0.04, 0.849	7 (16.6%)	35 (83.2%)	1.82, 0.177
SCLC (8)	3 (37.5) %	5 (62.5%)		5 (62.5%)	3 (37.5) %		2 (25%)	6 (75%)		4 (50%)	4 (50%)		4 (50%)	4 (50%)		2 (37.5%)	6 (62.5%)	

Association of target gene expression with sites of metastases

The most common site of metastases was the bones in 18 (36%) patients, followed by the liver in 10 (20%) patients, and adrenal was the least common site of metastases in three (6%) patients. There was a statistically significant relationship between HMGB3 (ΔCt) and the absence of bone metastases (χ²(1) = 4.78, p = 0.029), but there was no statistically significant relationship with CLCA2 (χ²(1) = 1.3, p = 0.254), ASH1 (χ²(1) = 0.05, p = 0.83), survivin (χ²(1) = 0.74, p = 0.39), CK7 (χ²(1) = 0.3, p = 0.585), and L587S (χ²(1) = 0.9, p = 0.342). In liver metastases also, there was no statistically significant association between HMGB3, ASH1, survivin, and L587S. In adrenal metastases, the results were insignificant between HMGB3, ASH1, survivin, CK7, and L587S. Only CLCA2 had higher expression in patients without adrenal metastases (p = 0.044).

## Discussion

In the current research, we utilized an RT-PCR-based assay for the identification of HMGB3, survivin, CLCA2, CK7, ASH1, and L587S gene expressions in lung cancer patients. These genes were selected in the present study as they are known to be expressed in patients with lung cancer.

The overall detection rate of the six target genes examined in lung cancer was in 42 (84%) patients testing positive. In eight (16%) of lung cancer patients, the target genes could not be detected in the PB samples. However, in patients without any gene expression, no common characteristics were identified. In a study conducted by Yu et al., similar to the current research, four genes were examined in 68 patients with adenocarcinoma lung: human telomerase reverse transcriptase (hTERT), CK-7, survivin, and thyroid transcription factor 1 (TTF-1) mRNA expression levels. The results showed that 82.5% of patients tested positive for all four markers, while 17.4% of patients tested negative [17]. The highest individual gene-positive detection rate was 58% of survivin, followed by CLCA2 in 56% and CK7 in 44% of patients. In Yu et al.'s study, hTERT was positive in 61.7% of patients, followed by survivin and CK7 in 41.8% of patients. In adenocarcinoma histology, the highest expression was of CLCA2 in 62%, followed by survivin in 57.1% of patients. In this research, in squamous cell carcinoma histology, survivin was most commonly expressed in 55% of patients, followed by CLCA2 and CK7 in 45% of patients each. In small cell carcinoma histology, survivin was the most commonly expressed in 80% of patients. L587S was more commonly expressed in small cell histology (40%) as compared to NSCLC. The least expressed gene was ASH1 in 25% of patients.

Westerman et al. studied ASH1 in SCLC patients and identified that SCLC had a thousand times more expression of ASH1 compared to NSCLC [18]. In the current study, ASH1 expression in NSCLC was in 13 (31.9%) patients as compared to SCLC in two (25%) patients.

Shinmura et al., in the immunohistochemistry analysis, reported very high expression of the CLCA2 gene in SCC lung in comparison to adenocarcinoma [19]. They further suggested that the CLCA2 gene, combined with other SCC-specific markers, may be utilized as a diagnostic marker for SCC lung. Contrary to this, in the present study, CLCA2 was expressed more in adenocarcinoma compared to SCC lung (62% versus 45%) and higher in stage IV in comparison to stage III. However, this was comparable to a study by Man et al. that found a 95.0% specificity in increased expression of CLCA2 in lung cancer. Similar to this work, Man et al. found that CLCA2's sensitivity and specificity in lung adenocarcinoma were 0.8% and 95.0%, respectively [20]. They hypothesized that CLCA2 might be a marker for lung cancer detection. Higher stages and nodal positive rates were linked to increased expression of HMGB3 in gastric cancer tissue, as reported by Tang et al. [21]. Song et al. reported that HMGB3 expression was higher in NSCLC than in non-malignant tissues and that it was associated with a worse prognosis. In the present research, patients with NSCLC (28.6%) and SCLC (37.5%) have overexpressed HMGB3. HMGB3 was identified as one of the predictive markers by Liu et al. after they performed proteogenomic characterization of SCLC patients [22].

Luo et al. observed the presence of CK7 and CK20 expression in lung cancer patients and proposed that there were variations in CK7 expression based on factors such as gender, age, lymph node involvement, grade, and invasiveness [23]. Additionally, CK7 expression had a prognostic impact on lung cancer patients. In this research, expression of CK7 was also high in males, aged> 50 years, smokers, and stage IV. Luo et al. mentioned in their study that expression levels did not correlate with staging, but in the current study, they had a statistically significant correlation with metastases. Contrary to Luo et al., CK7 expression was higher among smokers in this study, but the result was insignificant statistically. In an RT-PCR-based investigation, Monzo et al. discovered that, although survivin was overexpressed in NSCLC tissue, there was no discernible relationship between it and any of the following: age, gender, smoking, histopathologic subtype, differentiation, tumor, or nodal stage [24]. In the present study, the expression of survivin was the highest among all six target genes. It was expressed more in age > 50 years, smokers, and stage III. Similar to this work, Hirano et al. reported that smoking is linked to greater expression of nuclear survivin [25]. Additionally, squamous cell carcinoma expressed somewhat higher than adenocarcinoma.

L587S, a novel gene, is located on chromosome 18. It was identified through subtraction libraries to be specific to lung cancer [26]. In the current study, it was expressed more in SCLC than in NSCLC and in men and higher clinical stages.

Smokers exhibited greater relative target gene expression than non-smokers for each of the six target genes in the current research, indicating an association between smoking and both gene expression and carcinogenesis. Smoking has a substantial effect on lung carcinogenesis, as evidenced by the significantly greater expression of the NEK2, TTK, and PRC1 genes in smokers compared to non-smokers in lung cancer, according to research by Landi et al. [27].

In the current study, the proportion of females was smaller than that of the male subjects. Compared to females, males exhibited higher levels of the genes ASH1, CLCA2, CK7, and HMGB3. The study found that CLCA2 was more positive in patients over 50 years. Age had no significant association with HMGB3, survivin, and CK7. Survivin was most common in males, followed by CLCA2. Smoking had no significant relationship with HMGB3, CLCA2, survivin, and CK7. In stage III, CK7 was positive in 13 patients, while survivin was more positive in 17 patients in stage IV. ASH1, CK7, and HMGB3 expression were associated with higher stages but not with CLCA2, survivin, and L587S. Survivin was positive in 25 patients of NSCLC and CLCA2 in 23 patients. No significant association was identified with histology and HMGB3, CLCA2, ASH1, survivin, and CK7. Bone metastases were the most common site in 36% of patients, followed by liver in 20% and adrenal in 6%. There was a significant relationship between HMGB3 and the absence of bone metastases, but no significant relationship was found with CLCA2, ASH1, survivin, CK7, and L587S. Brain metastases showed no significant relationship with HMGB3, CLCA2, ASH1, survivin, CK7, and L587S. Only CLCA2 had an association without adrenal metastases.

Kim et al. mentioned that gender differences are due to sex hormones as they modulate gene expression [28]. Xiao et al. identified that male patients of lung adenocarcinoma had more burden of genetic alterations than female patients (male-to-female ratio = 1.636), and these alterations were associated with poor survival. Hence, these differences were responsible for variable outcomes [29]. Yuan et al. reported overexpression of estimated glomerular filtration rate (EGFR) in female patients compared to males and consequently more responsiveness to tyrosine kinase inhibitors [30]. Izbicka et al. used mass spectrometry to identify biomarkers in non-small cell lung cancer patients [31]. They measured 57 markers and concluded that, while a soluble cluster of differentiation 40 (sCD40) had prognostic value in females, biomarkers such as soluble Fas (sFAS), matrix metalloproteinase-9 (MMP-9), and plasminogen activator inhibitor-1 (PAI-1) were strongly predictive in males. Gender-specific endocrine variations were the cause of the observed alteration in gene expression. According to Whitehead et al., variations in gene expression can happen both within and between populations, emphasizing the variety of phenotypes [32]. Tabassum et al. suggested that a myriad of factors (e.g., geographical location, food habits, lifestyle, and ethnicity) can lead to variability in gene expression [33]. Thus, in the present study, lifestyle and geographical variation might be probable causes of variable gene expression as compared to other studies. In the present study, the overall percentage of positive detection was 84%, comparable to the 82.5 % reported by Yu et al., but higher by around 10% in the Sher et al. study. It has been suggested by Yoon et al. that the detection rate of CTC in PB may vary from 30% to 60% [34]. The expression of genes is heterogeneous, and even during tumor progression, there might be alternative gene expression; therefore, no tumor marker will be expressed consistently throughout all patients. Additionally, this limits single-marker reliability. The use of multiple gene markers may subdue tumor cell heterogeneity for marker expression and less number of CTC in blood.

The current study has certain limitations, including a small sample size and all patients having advanced cancer stages. Furthermore, we were unable to quantify CTCs in patients with lung cancer. Extended patient follow-up is necessary to assess gene expression in relation to treatment response, recurrence, and survival.

## Conclusions

CTCs can be a valuable tool for diagnosing and monitoring lung cancer patients. In addition to peripheral blood, this can be applied to other body fluids such as pleural effusion, bone marrow, and sputum. CTCs in peripheral blood can also be utilized for assessment of treatment response, prognosis, and follow-up to detect recurrence. However, further research is needed to determine the clinical significance of these markers, especially in larger groups of early-stage patients.
